# Gene Editing Strategies for Neurological and Mental Disorders: Advances in Delivery, Methodology, and Clinical Translation

**DOI:** 10.3390/cells15080720

**Published:** 2026-04-19

**Authors:** Amer Elias, Shani Stern

**Affiliations:** Sagol Department of Neurobiology, Faculty of Natural Sciences, University of Haifa, Haifa 3498838, Israel; aelias19@campus.haifa.ac.il

**Keywords:** gene editing, CRISPR-Cas, base editing, prime editing, neurological disorders, Central Nervous System (CNS), precision medicine, Adeno-Associated Virus (AAV), Blood–Brain Barrier (BBB), exosomes

## Abstract

**Highlights:**

**What are the main findings?**

- This review provides a comprehensive analysis of next-generation genome-editing tools, such as base editing and prime editing, which are specifically optimized for the post-mitotic environment of the central nervous system.
- It evaluates the evolving landscape of CNS delivery vehicles, contrasting traditional AAV limitations with advanced solutions like the AAVLINK recombination system for large cargo and the natural blood-brain barrier-crossing capabilities of stem cell-derived exosomes.
- The article catalogs pivotal clinical milestones, including the 2025 FDA approval of Itvisma for spinal muscular atrophy and the utilization of convection-enhanced delivery for Parkinson’s disease.
- Preclinical research highlights successful “genomic surgeries,” including the first systemic *in vivo* base editing for *MEF2C*-related autism spectrum disorder and high-fidelity prime editing to correct *ATP1A3* mutations in Alternating Hemiplegia of Childhood.
- Regulatory updates, such as the FDA Modernization Act 2.0, have accelerated the shift toward human-centric preclinical models, like iPSC-derived organoids and organ-on-chip technologies, as alternatives to traditional animal testing.

**What are the implications of the main findings?**

- Gene-targeted therapies represent a shift from symptomatic relief to permanent disease modification by addressing the underlying molecular etiology of neurological and psychiatric disorders.
- Because neurons are largely post-mitotic, the genomic edits introduced are not diluted over time, offering the potential for durable, lifelong therapeutic benefits from a single treatment.
- Breakthroughs in non-invasive delivery technologies, such as engineered exosomes and focused ultrasound, allow therapeutic agents to bypass the blood-brain barrier, which historically excluded 95% of treatments.
- The convergence of artificial intelligence, multi-omics, and systems biology will enable the development of high-precision, personalized genome editors with increased accuracy and reduced off-target risks.
- Enhanced early detection through newborn screening and next-generation sequencing allows for interventions during presymptomatic stages, potentially preventing irreversible neuronal loss.

**Abstract:**

Neurological and mental disorders are among the main causes of disability worldwide, affecting over three billion people and increasing the socioeconomic burden. Advances in molecular genetics and genome engineering have led to gene-targeted therapies that address root causes rather than just symptoms. This review covers current genome-editing tools, including CRISPR/Cas, base editing, and prime editing. The focus is on the benefits of gene editing in the central nervous system, where post-mitotic neurons allow lasting effects after a single treatment. It also discusses emerging delivery platforms such as viral vectors, nanoparticles, and exosome systems, as well as methods to bypass the blood–brain barrier. Recent clinical progress in spinal muscular atrophy, Parkinson’s disease, Huntington’s disease, and Alzheimer’s disease is highlighted, with promising preclinical results for autism, bipolar disorder, epilepsy, and other neurogenetic conditions. The review concludes with regulatory issues, market trends, and ongoing clinical trials, underscoring the potential of gene therapies to transform disease management and provide long-term solutions.

## 1. Introduction

Rare diseases, affecting fewer than 1 in 2000 people [1], impact about 473 million worldwide [2,3], with around 70% appearing in childhood [4]. Despite their prevalence, 90% of these diseases lack effective treatments [5]. Nearly half are neurological, and about 90% of childhood cases involve neurological issues [6]. Diagnosing neurologic disorders early remains difficult due to the absence of specific molecular markers, clear symptoms, and limited clinical expertise and therapies. However, recent advances in diagnostics, such as genomic next-generation sequencing, transcriptomics, and proteomics, have greatly improved diagnosis by providing functional insights often missed by genomic testing [7,8,9,10,11,12,13,14,15,16]. These innovations have shortened diagnostic times and increased identification rates. Additionally, more diseases are included in newborn screening (NBS) panels [17], allowing for the detection of presymptomatic patients and opening new avenues for early intervention and treatment.

Alongside improvements in early detection methods, a new wave of therapeutic innovations, including cutting-edge gene-targeted therapies, offers promising potential not only for managing symptoms but also for possibly changing the disease’s natural course. At present, there are 832 clinical trials focused on gene therapies for neurological conditions [18]. This evolving landscape underscores the urgent need to educate our clinical workforce to recognize and stratify patients who are suitable for these novel treatments. As therapies transition from trials into routine clinical practice and the pool of treatable patients expands, comprehensive education on gene therapies, treatment eligibility, and post-treatment care is critical. This article offers a detailed overview of available gene therapies for neurologic disorders, emphasizing their mechanisms, expected benefits, considerations for follow-up care, and ongoing preclinical and clinical trials.

Gene-targeted therapies represent a transformative class of medical interventions designed to treat or prevent disease by directly modifying, replacing, or modulating the expression of genetic material within a patient’s cells. Unlike conventional pharmacological approaches, which typically target downstream pathways, gene-targeted therapies address the underlying molecular etiology of a disorder. This broad category encompasses several core strategies: gene replacement (providing a functional copy of a defective gene), gene addition (supplementing therapeutic genes to counteract disease mechanisms), gene knockdown (using RNA interference or antisense oligonucleotides (ASOs) to silence harmful gene products), and gene editing (introducing permanent, targeted changes directly to the host genome). By addressing the root causes of genetic disorders, these treatments offer the potential for durable, one-time cures for previously incurable neurological and neuromuscular conditions.

### 1.1. Neurological Disorders: Scope and Impact Overview

Neurological disorders cover a wide range of conditions that affect the central and peripheral nervous systems. These include neurodegenerative diseases such as Alzheimer’s disease (AD) and Parkinson’s disease (PD), neurodevelopmental conditions like autism spectrum disorder (ASD), various types of epilepsy, and neuropsychiatric diseases such as bipolar disorder (BD) and schizophrenia. Together, they are the leading cause of disability globally. The 2021 Global Burden of Disease Study reports that about 3.4 billion people, roughly 43% of the world’s population, are impacted by these disorders, causing over 11.1 million deaths and 443 million disability-adjusted life years (DALYs) lost each year [19]. The overall burden has grown significantly over the past three decades, driven by aging populations and increased numbers worldwide [19]. Besides health implications, these chronic conditions create substantial economic and emotional challenges for individuals and society, often leading to permanent disability and a sharp decline in quality of life [19].

### 1.2. Genetic Basis

Gene editing is particularly suitable for neurology because many disorders, such as Huntington’s disease (HD) [20], spinal muscular atrophy (SMA) [21], Rett syndrome [22], ASD [23], and parts of Amyotrophic Lateral Sclerosis (ALS) [24] and PD [25], are monogenic. This straightforward genotype–phenotype link enables the development of programmable gene-editing tools that can directly target and correct harmful mutations or disable dangerous alleles [26]. Additionally, improvements in next-generation sequencing and the expansion of NBS panels have greatly reduced the time to diagnosis, enabling early intervention before irreversible neuronal loss occurs [7,8,9,10,11,12,13,14,15,16,17,27].

### 1.3. Durability

A key advantage of gene editing in the central nervous system (CNS) is its potential durability. Because neurons are largely post-mitotic cells that do not undergo cell division, genomic edits introduced into these cells are not diluted over time, allowing the correction to persist long term [28]. This raises the possibility that a single therapeutic intervention could provide lifelong benefits, representing a significant advantage over conventional treatments that require repeated dosing throughout a patient’s life. However, neurological disorders often involve additional cellular components beyond neurons. Some disease-associated mutations also affect glial cells [29], which retain proliferative capacity, as well as the brain extracellular matrix (ECM), which is produced by both neurons and glial cells and plays an important role in maintaining neural structure and signaling [30,31,32,33]. Despite this cellular complexity, neuronal dysfunction remains a major driver of many neurological phenotypes. Therefore, efficient and durable genetic correction in neurons is expected to provide substantial therapeutic benefit, even if additional cellular compartments may also contribute to disease pathology.

### 1.4. Cell-Type Specificity

The field has advanced to enable high cell-type specificity, leveraging specialized promoters and engineered delivery vehicles to restrict editing to disease-relevant populations. For instance, promoters can be used to selectively target dopaminergic neurons in PD or GABAergic interneurons in Dravet syndrome [34]. Innovative systems like the Ribonucleoprotein Delivery system (RIDE), a customizable virus-like particle, further enhance this specificity through programmable cell tropism, which can be customized with single-chain antibodies to target specific neural subpopulations while minimizing off-target effects in non-target organs [35].

### 1.5. Multimodal Potential

Gene editing offers vast multimodal potential that extends beyond simple DNA correction, including gene silencing, which disrupts the expression of toxic gain-of-function alleles (such as mutant huntingtin or alpha-synuclein) [36,37], risk allele modulation, where we downregulate risk factors like APOE ε4 in AD to shift cellular metabolism toward a neuroprotective state [38,39], and functional modulation that utilize CRISPRa (activation) to upregulate compensatory genes or CRISPRi (interference) to repress transcriptional repressors, effectively rewiring dysfunctional cellular pathways [40].

### 1.6. Therapeutic Strategies Combination

Gene editing techniques are highly complementary to other treatments and could have synergistic effects when used together. They can be combined with gene-supplementation methods, such as RNA-targeting drugs, or ASOs, to improve therapeutic outcomes [41,42]. For instance, combining an Adenine Base Editor (ABE) with an ASO has demonstrated superior outcomes compared with ASO alone in animal studies [43]. Additionally, combining gene therapy with conventional drugs like L-DOPA in Parkinson’s disease or Riluzole in ALS could provide more comprehensive approaches for managing progressive neurodegenerative conditions [44,45].

## 2. Targeted Gene Therapy

### 2.1. Genetic Materials

The genetic material utilized in targeted gene therapies varies depending on the therapeutic objective and the specific tool being deployed. Deoxyribonucleic acid (DNA) is frequently used in the form of plasmids (circular DNA) or complementary DNA (cDNA) to replace or supplement missing or defective genes [46]. In the case of viral delivery, this DNA often resides in the cell nucleus as episomes (circularized molecules that do not integrate into the host genome), allowing long-term expression in non-dividing cells such as neurons [47,48]. Ribonucleic acid (RNA) is another vital component, including messenger RNA (mRNA) for the transient expression of therapeutic proteins or genome-editing enzymes, and small non-coding RNAs, such as siRNA or miRNA, used for gene silencing [49,50,51]. Furthermore, ASOs are short, synthetic single-stranded DNA or RNA chains designed to bind specific mRNA sequences to trigger degradation or modulate splicing [52,53,54].

### 2.2. Mechanisms of Action for Gene-Editing Tools

The development of programmable nucleases has created a flexible toolkit for precise genome editing by enabling targeted DNA cuts at specific genomic sites. Early versions include Zinc Finger Nucleases (ZFNs) and Transcription Activator-Like Effector Nucleases (TALENs), which both depend on engineered DNA-binding protein domains fused to the FokI endonuclease’s catalytic domain [55,56]. ZFNs are made up of zinc finger motif arrays, each recognizing a particular DNA triplet through amino acid interactions with the DNA’s major groove. These modules are combined to target longer DNA sequences, usually 9–18 base pairs. Since FokI operates as a dimer, two ZFN monomers must bind to opposite strands with proper spacing to enable FokI dimerization and produce a site-specific double-strand break (DSB). Designing ZFNs often involves complex protein engineering and meticulous optimization to ensure high specificity and minimize off-target effects [57,58].

TALENs are based on transcription activator-like effector proteins originally derived from Xanthomonas bacteria. Their DNA-binding domain contains tandem repeats of approximately 34 amino acids, each recognizing a single nucleotide through two hypervariable residues known as repeat-variable diresidues (RVDs). This one-to-one nucleotide recognition code simplifies the design of TALENs compared with ZFNs. Similar to ZFNs, TALEN DNA-binding domains are fused to the FokI nuclease domain, and cleavage occurs only when two TALEN monomers bind to adjacent DNA sites and allow FokI dimerization to generate a DSB. Although TALENs are generally easier to design and often exhibit high targeting specificity, their large size can make delivery, particularly via viral vectors such as AAV, more challenging [57,58].

**Figure 1:**
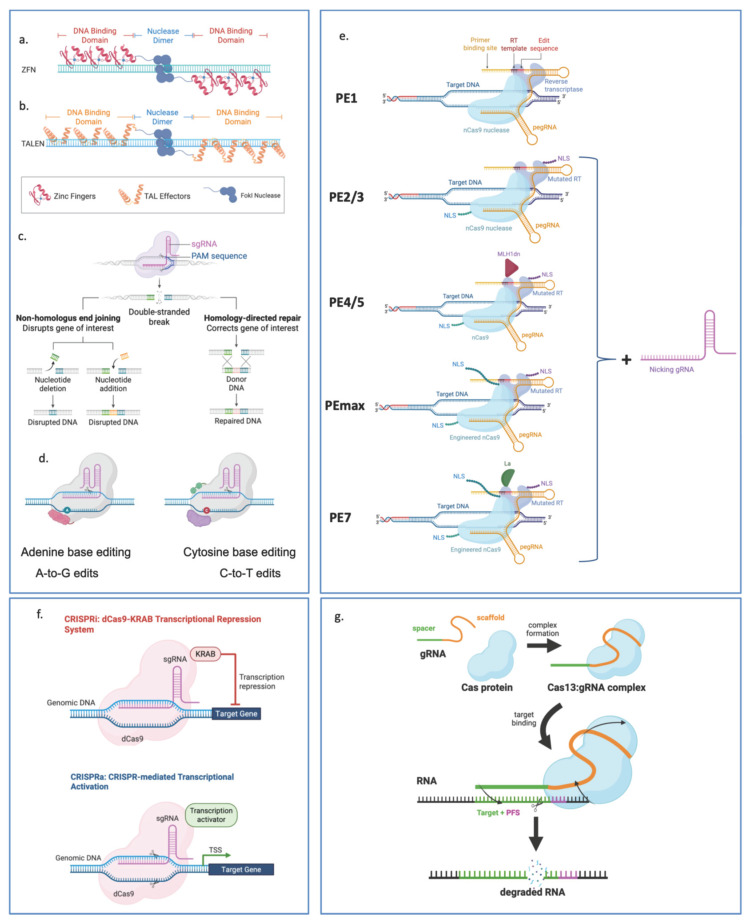
Gene editing tools. (**a**) Zinc Finger Nucleases (ZFNs): Engineered DNA-binding domains fused to a FokI nuclease dimer, requiring two subunits to bind adjacent sites to create a double-strand break. (**b**) Transcription activator-like effector nucleases (TALENs): Similar to ZFNs but utilizing TALE repeat domains for DNA recognition, providing modularity for targeting specific sequences. (**c**) CRISPR-Cas9: An RNA-guided Cas9 endonuclease creates a DSB at a site complementary to the sgRNA near a PAM. (**a**–**c**) causes a DSB. The break can be repaired by host mechanisms such as NHEJ, which is error-prone and can cause gene disruption via indels, or HDR, which allows precise gene editing when a donor template is provided. (**d**) Adenine base-editing and Cytosine base-editing: Fusion of a nCas9 to a deaminase enzyme. Adenine Base Editors convert A•T to G•C, while Cytosine Base Editors convert C•G to T•A without requiring DSBs. (**e**) The evolution of PE: PE1 consists of wild-type M-MLV RT fused to the C-terminus of nCas9. PE2 was obtained by the introduction of five-point mutations into the M-MLV and adding a nuclear localization signal (NLS). Further incorporation of an additional sgRNA to nick the complementary strand produced PE3. Advancement of PE with cellular factors includes co-expressing the dominant-negative MLH1 mutant (MLH1dn) with PE2 or PE3, generating PE4 and PE5, and mitigating MMR effects. PEmax was generated by codon-optimization of the M-MLV RT of PE2, adding an amino acid linker that includes additional SV40 NLS between nCas9 and M-MLV RT, and introducing R221 and N394 K mutations into nCas9. PE7 is created by fusing the RNA-binding protein La to the C-terminus of nCas9 in PEmax, further increasing editing performance. (**f**) CRISPRi and CRISPRa: Utilization of dCas9, CRISPRi uses repressor domains (e.g., KRAB) to silence genes, while CRISPRa uses transcriptional activators to upregulate target genes. (**g**) CRISPR-Cas13: A specialized system where the Cas13:gRNA (or CasRx:gRNA) complex targets and degrades specific RNA molecules rather than genomic DNA. Created by BioRender.com.

More recently, the CRISPR-Cas9 system has revolutionized genome editing due to its simplicity and programmability. Derived from a bacterial adaptive immune system, CRISPR-Cas9 uses a short single-guide RNA (sgRNA) to direct the Cas9 nuclease to a complementary genomic sequence adjacent to a Protospacer Adjacent Motif (PAM), typically the NGG sequence recognized by Streptococcus pyogenes Cas9 [59,60]. The sgRNA contains a 20-nucleotide spacer sequence that base-pairs with the target DNA, allowing Cas9 to bind and induce a DBS through its two nuclease domains: the HNH domain, which cleaves the DNA strand complementary to the guide RNA, and the RuvC domain, which cleaves the non-complementary strand. Following cleavage, the DSB is repaired by endogenous cellular DNA repair pathways. The Non-Homologous End Joining (NHEJ) pathway ligates the DNA ends directly but is error-prone, frequently introducing insertions or deletions (indels) that can disrupt gene function and generate knockout mutations. Alternatively, the Homology-Directed Repair (HDR) pathway can be used for precise genome editing when a donor DNA template containing homologous sequences is supplied, enabling the introduction of specific nucleotide substitutions, insertions, or gene corrections [61,62] (Figure 1c).

Beyond this, newer approaches have emerged. Base Editing (BE) employs a catalytically inactive Cas9 (dCas9) or a Cas9 nickase (nCas9) fused to a deaminase enzyme to chemically convert one DNA base into another (e.g., C-to-T or A-to-G) without inducing DSBs, thus reducing the risk of unintended genomic rearrangements [63,64] (Figure 1d). Prime Editing (PE), described as a versatile “search-and-replace” tool, combines a Cas9 nickase fused to a reverse transcriptase (RT) with a prime editing guide RNA (pegRNA). The pegRNA guides this complex to the target site and serves as a template for the RT to directly incorporate the desired DNA changes, including all 12 types of base-to-base conversions, enabling precise substitutions, small insertions, or deletions [65] (Figure 1e). PE has rapidly evolved from its initial proof-of-concept into a sophisticated toolkit through several key iterations designed to bypass cellular bottlenecks and delivery constraints. The original PE1 system established the described “search-and-replace” mechanism. PE2 significantly boosted this efficiency (up to 5.1-fold) by introducing five specific mutations into the RT to improve its catalytic activity and thermostability. PE3 further enhanced performance by adding a second “nicking” guide RNA to the unedited strand, which tricks the cell into using the edited strand as a repair template [66,67,68] (Figure 1e). Subsequent advancements addressed the cell’s own defense mechanisms; PE4 and PE5 (derivatives of PE2 and PE3) incorporate an engineered protein (MLH1dn) to temporarily inhibit the DNA mismatch repair (MMR) pathway, which otherwise tends to “fix” and remove the desired edits (Figure 1e). Most recently, PEmax optimized the protein architecture with improved nuclear localization and codon usage, while PE6 and PE7 introduced highly compact RTs and protective proteins like La to stabilize the guide RNA (Figure 1e). These iterations have collectively moved the technology from low-efficiency laboratory tests toward robust clinical applications, including the first human trials for chronic granulomatous disease in 2024 [66,67,69,70].

Another set of epigenomic editing tools is CRISPRi and CRISPRa, which use dCas9 fused to transcriptional repressors or activators, respectively. Instead of cutting DNA, they bind to promoters or regulatory regions to suppress or enhance gene expression [71,72] (Figure 1f). Lastly, RNA editing tools such as Cas13 and CasRx target mRNA rather than DNA [73,74] (Figure 1g). They use RNA-guided nucleases to specifically degrade or modify transcripts, offering a reversible, transient alternative to permanent genomic edits [75].

### 2.3. Limitations and Safety Challenges in Neuro-Gene Editing

The shift of gene editing from a research tool to a clinical solution for neurological and psychiatric disorders faces significant biological and technical challenges. Unlike regenerating tissues, the CNS mainly consists of postmitotic neurons that rarely divide. This lack of cell turnover means that any errors or adverse reactions from editing are permanent, making the brain highly sensitive to mistakes. Consequently, there is a very small margin for error, requiring a careful assessment of the risks associated with genomic surgery in the brain [76].

#### 2.3.1. Off-Target Modifications and Bystander Effects

A key safety concern is off-target activity, in which tools like the Cas9 nuclease cut DNA at unintended sites that resemble the target sequence. In long-lived neurons, such mutations could lead to genomic instability or even cause cancer [77]. Besides these typical off-target effects, researchers must also consider bystander effects, which involve unintended changes to nearby bases within the “activity window” of base editors and prime editors or large chromosomal rearrangements [78]. Since psychiatric disorders often involve complex regulatory regions, an unintentional edit in a neighboring enhancer or promoter might accidentally disrupt the regulation of genes important for cell functionality [79].

To address and evaluate these risks, several advanced strategies are being integrated into the therapeutic development process. High-fidelity Cas9 variants like HiFi-Cas9 and Sniper-Cas9 are engineered with structural changes to reduce their tendency to target mismatched DNA sequences [80,81]. The field is also moving away from relying solely on in silico predictions toward using experimental, genome-wide detection tools such as GUIDE-seq, CIRCLE-seq, and Digenome-seq [82,83,84]. These techniques facilitate the pinpointing of actual cleavage sites in human neuronal cell lines before moving to in vivo models. Additionally, transient delivery approaches, using mRNA or ribonucleoproteins (RNPs) instead of permanent viral vectors, help minimize the risk window by ensuring the editing components are broken down soon after the intended modification [85].

#### 2.3.2. Long-Term Stability and Potential Side Effects

While these technical advances improve immediate precision, the brain’s limited regenerative capacity means that the long-term stability of gene editing remains both a clinical achievement and a significant risk. Once a genetic change occurs, there is currently no “off-switch” or way to undo it if adverse effects appear years later [78,86]. A major concern is the immunogenicity of the editing tools themselves; prolonged exposure to bacterial proteins from AAV vectors can lead to sustained neuroinflammation [87]. In many psychiatric conditions where low-level neuroinflammation is suspected to contribute, introducing a persistent immune-stimulating protein could worsen symptoms that the therapy is aimed to address [88]. Additionally, the complexity of psychiatric genetics raises the risk of unintended functional side effects. Genes like DISC1 and BDNF, which are highly pleiotropic, serve different biological roles at various stages of life [89,90]. Consequently, permanent modifications in adults could have unforeseen impacts on neural plasticity or neurogenesis that manifest only with age [91]. To mitigate these long-term risks, developing sensitive, non-invasive monitoring methods, such as longitudinal PET imaging to track protein expression over time and fluid biomarkers in blood or cerebrospinal fluid to detect early signs of genomic instability or cellular stress, is essential [92].

### 2.4. Delivery Technologies and Mechanisms

Delivery technologies are specialized vehicles or methods that transport genetic material or editing tools into target cells and tissues [93]. These systems are designed to protect nucleic acid cargo from degradation by nucleases during circulation and cellular uptake [94]. Efficient delivery is a critical determinant of gene therapy success because the therapeutic payload must reach the appropriate intracellular compartment to exert its function [95]. An important characteristic of these delivery platforms is their packaging or payload capacity, which defines the maximum size of genetic material that can be transported and therefore constrains the complexity of therapeutic constructs that can be introduced into cells [96,97].

#### 2.4.1. Viral Delivery Systems and Capacity Constraints

Viral delivery systems are the most established methods for delivering therapeutic genetic material into target cells, especially in the CNS [46,98]. They work by exploiting the natural infection processes of viruses, in which their replication-related genes are removed and replaced with a therapeutic DNA or RNA payload, rendering the vector unable to replicate [99]. These vectors are highly effective at transducing both dividing and non-dividing cells [46]. However, a key challenge is the limited capacity of each viral capsid. The size of the therapeutic sequence, including promoters, regulatory elements, and the transgene, must stay within the virus’s physical packaging limit to ensure successful assembly and strong transgene expression [100].

**Figure 2:**
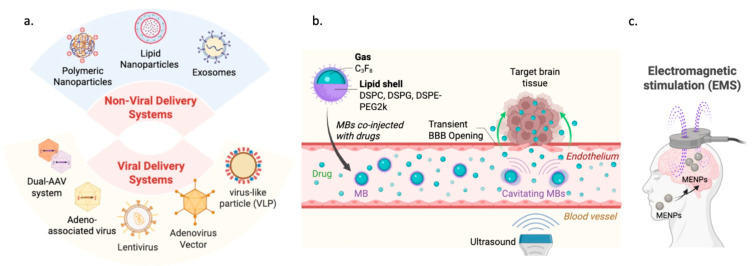
Delivery strategies to improve CNS-targeted therapeutics include both viral and non-viral systems. (**a**) Viral vectors, including AAV, lentivirus, adenovirus, dual-AAV systems, and VLPs, are commonly used for effective gene transfer, leveraging viral entry mechanisms to deliver genetic material into both dividing and non-dividing cells, allowing for stable or transient expression of therapeutic genes. On the other hand, non-viral platforms like LNPs, polymeric nanoparticles, and exosomes provide versatile options with higher payload capacity and lower immunogenicity. These carriers, whether synthetic or biologically derived, encapsulate nucleic acids or ribonucleoprotein complexes and promote cellular uptake via endocytosis or membrane fusion. Additionally, advanced physical and magnetically guided techniques delivery systems for CNS penetration are used, including (**b**) focused ultrasound-induced BBB opening involves administering microbubbles alongside therapeutic agents, then applying targeted ultrasound to cause a temporary, localized disruption of the BBB. The acoustic cavitation of microbubbles creates mechanical forces that briefly loosen endothelial tight junctions, permitting drugs or gene-editing cargos to pass into brain tissue. This method allows for non-invasive, region-specific delivery while maintaining the overall integrity of the BBB. (**c**) Magnetically guided nanoparticle delivery involves using magnetoelectric nanoparticles that can traverse the BBB when external magnetic fields or electromagnetic stimulation are applied. These particles allow precise targeting of gene-editing payloads to specific brain areas without permanently opening the BBB. Moreover, electromagnetic stimulation can boost cellular uptake and improve the accuracy of the delivery. Created by BioRender.com.

Adeno-Associated Virus (AAV) is the most commonly used vector for in vivo CNS studies because it is nonpathogenic and can maintain long-term expression in postmitotic neurons [98,101] (Figure 2a). However, its packaging capacity is limited to approximately 4.5–4.7 kb, making it challenging to accommodate traditional CRISPR-Cas9 systems. For example, the SpCas9 gene alone is around 4.1 kb, leaving minimal space for essential promoters and regulatory sequences. Consequently, researchers often choose smaller Cas orthologs, such as SaCas9, or use split/dual-AAV systems to fit within the vector [100,102].

In contrast, lentiviral vectors can carry larger payloads of 8 to 10 kb, with some designs accommodating up to 18 kb, allowing the delivery of full-length genes or multiple editing components in a single vector (Figure 2a). While they enable persistent expression through integration into the host genome, their larger size can restrict their distribution within the brain [103,104]. Adenoviral vectors (AdV), originally used in gene therapy, have an approximate capacity of 8 kb. Although they can transport large genes, their clinical use in CNS treatments is limited by their high immunogenicity and potential to trigger inflammatory responses [46,105].

#### 2.4.2. Non-Viral Delivery Systems

Non-viral delivery systems are synthetic or physical platforms designed to overcome the limitations of viral vectors, such as their small packaging capacity, risk of integrating into the genome, and pre-existing immunity [106,107]. These systems are highly valued for their ability to carry larger and more diverse payloads, often surpassing the 4.7 kb limit of AAV vectors, enabling the delivery of extensive genetic elements, such as full-length genes or complex prime editing tools. A key safety benefit of non-viral methods is their transient nature; they typically deliver cargo as mRNA or ribonucleoprotein (RNP) complexes that degrade within days inside the cell. This limited duration ensures editing tools are active only as long as needed for the desired modification, reducing the risk of off-target effects and preventing insertional oncogenesis. Additionally, these synthetic vehicles tend to elicit weaker immune responses than viral capsids, making repeat dosing feasible if necessary [108]. Non-viral delivery is generally classified into chemical approaches, such as Lipid Nanoparticles (LNPs) and polymers, and physical techniques, including electroporation used in ex vivo cell therapy and microinjection [107].

##### Lipid Nanoparticles

The most commonly used non-viral delivery tool is LNPs, which encapsulate nucleic acids within a protective lipid bilayer and are distinguished by their substantial payload capacity (Figure 2a). Their relatively large physical dimensions, typically 50–150 nanometers and occasionally up to 500 nanometers, enable them to convey genetic material that exceeds the capacity of viral vectors [109,110,111]. Post-delivery, LNPs are readily degraded by physiological processes, thereby enhancing safety by constraining the duration of genome editing activity [112]. While highly efficient for hepatic targeting, LNPs’ large size poses a significant obstacle to their traversal of the blood–brain barrier (BBB) [113,114].

##### Polymeric Nanoparticles

An additional method is Polymeric Nanoparticles (PNPs), which are composed of various cationic nanomaterials such as poly-L-lysine, poly-L-ornithine, chitosan, or gelatin [115] (Figure 2a). These particles typically range in size from 10 to 200 nanometers and serve as versatile delivery platforms, with payload capacity contingent on the specific nanomaterials employed. This adaptability facilitates the transport of diverse genetic materials, including plasmids, RNA, or oligonucleotides. A primary advantage of PNPs is their safe and easy preparation, offering a non-pathogenic alternative that can be administered at higher, more effective doses than viral vectors [116,117]. While PNPs can be customized with surface ligands, stealth coatings, or targeting molecules to improve BBB penetration and cell-specific uptake, they are often limited by a relatively low delivery and transduction efficiency compared to viral systems [117].

##### Exosomes

Exosomes, natural membrane-bound vesicles ranging from 30 to 150 nm, represent a sophisticated “cell-free” therapeutic platform characterized by high biocompatibility, inherent stability, and minimal immunogenicity [118,119] (Figure 2a). Functioning as essential mediators of intercellular communication, these vesicles can be bioengineered to transport a versatile array of biomolecules, including mRNA, miRNA, siRNA, and even functional CRISPR-Cas9 ribonucleoprotein complexes [120,121,122,123,124]. A primary advantage of exosome-based delivery for neurological indications is the ability of these particles to naturally cross the BBB, facilitating non-invasive access to neural tissue [125,126,127]. Preclinical studies have highlighted their robust therapeutic potential; for instance, intranasal administration of stem cell-derived extracellular vesicles (EVs) has been shown to improve motor symptoms and normalize tyrosine hydroxylase expression in PD models [128,129,130], while Mesenchymal stem cell-derived exosomes containing antioxidant miRNAs provide significant neuroprotection to motor neurons [131,132]. Further research demonstrated that exosomes from healthy stem cells (MSCs and iPSCs) successfully reduce cellular hyperexcitability and promote normal maturation in human iPSC-derived cortical neurons with SHANK3 mutations. Additionally, intranasal delivery of these healthy iPSC-derived exosomes notably improves ASD-related behavioral issues, particularly emotion recognition (emotional state preference), in Shank3B −/− mouse models [133]. Despite this promise, broad clinical translation remains hindered by technical hurdles in large-scale manufacturing, the complexity of high-purity isolation, and the need for precise ligand engineering to ensure selective targeting of specific neuronal populations [134,135].

#### 2.4.3. Advanced Delivery Systems for CNS Penetration

The CNS remains one of the most challenging targets for gene therapy due to the restrictive nature of the BBB, which prevents approximately 95% of therapeutic agents from reaching the brain tissue [136]. To overcome this obstacle, advanced delivery systems have been developed to bypass the BBB while also addressing the limitations of traditional vectors [137].

For instance, the AAVLINK strategy tackles the 4.7 kb packaging limit of AAVs by employing Cre/lox-mediated intermolecular DNA recombination. In this approach, a long therapeutic gene is split into segments that are separately packaged into multiple AAVs (Figure 2a); upon entry into the target cell, these segments recombine to reconstitute the full-length gene, enabling the delivery of genes exceeding 11 kb, such as those involved in autism (Shank3) or epilepsy (SCN1A) [138,139].

An additional approach is RIDE, which uses biosynthetic virus-like particles (VLPs) to deliver transient ribonucleoprotein (RNP) complexes (Figure 2a). These VLPs have a payload capacity comparable to lentiviral vectors but allow for “hit-and-run” editing, as the Cas9 protein degrades within 72 h, thereby substantially reducing off-target effects and immunogenicity [35]. Furthermore, engineered AAV capsids, such as AAV-PHP.eB, created through directed evolution, demonstrate enhanced ability to cross the BBB with greater efficiency than natural serotypes, permitting systemic administration at lower, safer doses [93,140].

Physical and magnetically guided techniques also contribute to CNS delivery; for example, Focused Ultrasound (FUS) combined with microbubbles (MBs) can transiently open the BBB to facilitate the passage of therapeutic particles [141,142,143] (Figure 2b), while magneto-electric nanoparticles (MENPs) can be directed across the BBB using magnetic fields to deliver CRISPR components without disrupting cellular junctions [144,145] (Figure 2c).

The overarching challenge remains a fundamental trade-off between vector size and capacity: smaller vectors such as AAV, which are approximately 25 nm in size, are more efficient at crossing the BBB but lack the necessary capacity for advanced tools like prime editors; conversely, larger systems like LNPs offer the capacity required for next-generation gene editing but encounter significant barriers in penetrating the BBB.

## 3. Disease-Specific Landscape and Clinical Translation (Table 1)

Neurological Disorders: From Rescue to Modification

The clinical landscape for neurological gene therapy has pivoted from proof-of-concept to systemic and localized optimization.

### 3.1. Spinal Muscular Atrophy

In November 2025, the therapeutic paradigm for SMA expanded with the FDA approval of onasemnogene abeparvovec-brve (Itvisma). This treatment is a non-replicating recombinant gene therapy that utilizes an AAV9 capsid as a delivery vehicle. The vector carries a functional human survival motor neuron 1 (*SMN1*) transgene, whose expression is driven by a constitutive cytomegalovirus (CMV)-enhanced chicken β-actin hybrid promoter to ensure continuous, sustained production of the essential SMN protein within motor neurons. Specifically indicated for patients aged 2 years and older, this intrathecal formulation is administered via a single lumbar puncture into the cerebrospinal fluid. Unlike its intravenous predecessor, this concentrated delivery route uses a fixed dosing regimen independent of patient weight, significantly reducing the total viral load required and mitigating the risk of systemic complications, such as hepatotoxicity, while ensuring robust transgene expression directly within the spinal cord parenchyma [146,147].

### 3.2. Parkinson’s Disease

Current therapeutic approaches have pivoted toward disease-modifying neuroprotection and restorative cell replacement, moving beyond the transient symptomatic relief provided by traditional levodopa-based regimens [45].

AAV-Mediated Neuroprotection (AB-1005): Starting in September 2025, the Phase 2 REGENERATE-PD trial is evaluating AB-1005, a recombinant gene therapy using an AAV2 vector, with 132 participants. This construct is engineered to express human Glial Cell Line-Derived Neurotrophic Factor (*GDNF*), a potent neurotrophic factor that binds to the GFRα1/RET receptor complex, triggering downstream pro-survival signaling pathways, specifically PI3K/Akt and MAPK/ERK, within dopaminergic neurons. Delivery is achieved through a single convection-enhanced delivery (CED) procedure, a pressurized infusion technique that ensures the homogeneous distribution of AAV2-GDNF throughout the putamen. By establishing a localized “bio-factory” for continuous GDNF secretion, this intervention aims to stabilize the nigrostriatal circuitry and promote the functional recovery of surviving neurons [148].

Autologous Ex Vivo Gene Therapy (exPDite-2): Complementing these direct viral approaches is the exPDite-2 Phase 3 trial, which involves approximately 102 participants and reached a clinical milestone with the first patient treated in September 2025. This trial utilizes an autologous cell-based platform in which the patient’s own cells are harvested and genetically modified ex vivo to overexpress specific therapeutic factors (such as Aromatic L-amino acid decarboxylase, AADC) before reimplantation into the striatum. This autologous transplantation strategy aims to restore the brain’s enzymatic capacity to convert levodopa into dopamine locally, while minimizing the risk of immunogenic rejection or the inflammatory MRI signals observed in previous allogeneic or high-dose viral trials. By precisely calibrating the dopamine synthesis machinery at the circuit level, exPDite-2 seeks to provide a durable, long-term correction of motor fluctuations [149].

### 3.3. Huntington’s Disease

Precision silencing of the huntingtin (*HTT*) gene remains the primary objective for treating this autosomal dominant trinucleotide repeat disorder. The investigational therapy AMT-130 represents a first-in-class recombinant AAV5-based vector engineered to deliver a specialized microRNA (miRNA) expression cassette. This cassette is designed to undergo endogenous processing into an active miRNA that targets and binds to a conserved sequence on the human HTT mRNA via the RNA-induced silencing complex (RISC). By facilitating the catalytic degradation of both mutant and wild-type HTT transcripts, the therapy aims to significantly reduce the concentration of the toxic, misfolded huntingtin protein and its associated intranuclear aggregates. Administered via a single, MRI-guided CED into the striatum (putamen and caudate), AMT-130 transforms local neurons into persistent inhibitory units. While in Phase 1/2 evaluation, the regulatory trajectory encountered a hurdle in late 2025 when the FDA requested longitudinal data beyond the initial cohorts to validate the durability and safety of long-term non-selective huntingtin suppression before proceeding with a Biologics License Application (BLA) [150,151]. An additional trial is investigating SPK-10001, a one-time AAV-based gene therapy developed by Spark Therapeutics that delivers microRNA that specifically targets and degrades the HTT mRNA, lowering mHTT protein levels. It is delivered directly into the brain via surgical infusion and is currently in Phase 1/2 clinical trials to evaluate its safety and potential to halt disease progression [152]. A recent Phase 1/2 trial involving 29 patients is examining AB-1001 (formerly BV-101), a gene therapy based on AAVrh10 developed by Brainvectis for early Huntington’s disease. The treatment consists of a single surgical infusion into the brain that delivers the CYP46A1 gene. This gene helps break down brain cholesterol into 24-hydroxycholesterol (24OHC), promoting neuron health, restoring cholesterol metabolism, and reducing the toxic mutant huntingtin protein, which may slow the disease’s progression [153].

### 3.4. Alzheimer’s Disease

While clinical efforts primarily target monoclonal antibodies against amyloid-beta (Aβ) and tau [154,155], research in 2025 has shifted towards gene therapy approaches that modulate genetic risk factors at the transcriptional level [156]. A current study is testing the LX1001 treatment, which focuses on AAV-based delivery of the neuroprotective apolipoprotein E2 (APOE2) or on CRISPR-mediated disruption of the pro-inflammatory APOEε4 allele [157,158]. This approach uses AAVrh.10, which are renowned for targeting neurons effectively, and are administered via intracisternal or intraparenchymal injections to achieve widespread distribution in the cortex and hippocampus. By employing neuron-specific promoters like synapsin I, these therapies promote *APOE2* expression, which competes with APOE4 for receptor binding and lipid transport. This process enhances amyloid clearance and reduces microglial-driven neuroinflammation. Ultimately, these interventions aim to shift the brain’s biochemical environment from neurodegeneration toward neuroprotection, targeting the underlying genetic causes rather than just removing protein debris [159,160,161].

An additional trial, in phase 1, is testing an AAV2 vector that delivers the Brain-Derived Neurotrophic Factor (*BDNF*) gene directly into the brain. The trial plans to enrol 12 participants total, 6 with early AD and 6 with Mild Cognitive Impairment (MCI). It aims to test whether continuous BDNF production can slow or reverse disease progression in early AD and MCI patients [162]. Initial reports indicate the therapy has been safe, with no serious adverse events related to the procedure, and it has shown signs of restoring brain activity in treated areas [163].

**Table 1:** Ongoing Gene Therapy Clinical Trials (2025–2026).

Disorder	Therapy/Vector	Phase	Status	Reference
SMA	Itvisma (OAV101 IT)	Approved	Post-market monitoring (approved November 2025)	[146]
Parkinson’s disease	AB-1005 (AAV2-GDNF)	Phase 2	Safety & Efficacy; Enrolling in EU/US	[148]
Parkinson’s disease	exPDite-2 (Autologous)	Phase 3	First patient treated September 2025	[149]
Huntington’s disease	AMT-130 (AAV5-miRNA)	Phase 1/2	FDA feedback pending for BLA path	[150,151]
Huntington’s disease	SPK-10001 (Engineered AAV-miRNA)	Phase 1/2	Evaluate the Safety, Tolerability, and Efficacy	[152]
Huntington’s disease	AB-1001 (AAVrh10-*CYP46A1*)	Phase 1/2	Dose Finding Study to Evaluate the Safety, Tolerability, and Efficacy	[153]
Alzheimer’s disease	LX1001 (AAVrh.10hAPOE2)	Phase 1/2	Safety evaluate	[158]
Alzheimer’s disease	AAV2-BDNF	Phase 1	Safety evaluate	[163]

## 4. Preclinical Studies That Harness Novel Gene Editing Tools to Treat ASD, Bipolar Disorder, Parkinson’s Disease, Alternating Hemiplegia, and Dravet Syndrome (Table 2)

The preclinical gene therapy landscape has evolved to focus on next-generation genome editing, particularly BE and PE. These technologies are well-suited for the CNS because they enable the correction of harmful variants in post-mitotic neurons without creating DSBs. They greatly reduce the likelihood of random indels, extensive genomic deletions, or chromosomal translocations, which are common issues with standard CRISPR-Cas9 approaches.

### 4.1. Autism Spectrum Disorder: In Vivo Base Editing of MEF2C

A landmark 2024 study effectively used ABEs to fix a point mutation in the Myocyte Enhancer Factor 2C (*MEF2C*) gene, a key regulator of cortical structure and synaptic plasticity. Delivered through an engineered AAV-PHP.eB capsid for widespread brain distribution, the AeCBE system enabled the hydrolytic deamination of a specific adenine, successfully reversing the mutation responsible for *MEF2C* haploinsufficiency. By restoring natural protein levels in the prefrontal cortex and hippocampus, the therapy reestablished excitatory–inhibitory synaptic balance and reduced core ASD-like behaviors, such as social avoidance and repetitive grooming. This marks the first successful use of systemic in vivo BE to treat a complex neurodevelopmental disorder [164].

Additional preclinical studies targeting CHD8-ASD have explored the use of CRISPRa to restore gene expression in cases of haploinsufficiency. *CHD8* encodes a chromatin remodeler that plays a central role in neurodevelopment, including regulation of Wnt signaling, neuronal proliferation, and synaptic gene networks, and loss-of-function mutations in this gene are among the most strongly associated genetic causes of ASD [165,166]. In preclinical models, including human iPSC-derived neurons and brain organoids carrying CHD8 mutations, CRISPRa-mediated upregulation of the remaining functional allele has been shown to partially restore CHD8 expression levels and rescue downstream transcriptional dysregulation [167]. These corrections were associated with improvements in neuronal phenotypes, including normalization of gene expression programs, synaptic function, and neuronal morphology. Importantly, this approach avoids permanent genome modification while addressing the dosage sensitivity characteristic of CHD8-related ASD. Together, these studies support CRISPRa-based gene activation as a promising therapeutic strategy for haploinsufficient neurodevelopmental disorders, although further in vivo validation and optimization of delivery systems remain necessary before clinical translation.

### 4.2. Bipolar Disorder and Affective Pathologies: CRISPR-SKIP and Epigenetic Modulation

Research into psychiatric gene modulation is currently focused on epigenetic editing and transcriptional tuning rather than genomic correction, due to the polygenic nature of these disorders. Tools such as CRISPR-SKIP are precise gene-editing methods that employ base editors to intentionally omit specific exons during RNA splicing. They achieve this by altering a single DNA base at a splice acceptor or donor site, disrupting splicing without generating large, potentially risky DSBs [168]. Such a tool can be employed to alter key splice donor or acceptor sites, such as inducing transitions, which have the potential to allow cells to bypass pathogenic splice-site mutations, as in the exon 38 splice donor of the *UNC13B* gene, and the c.393G>A mutation in exon 2 of the *NLRP3* gene [168]. Additionally, epigenetic editing with dCas9-VPR (CRISPRa) or dCas9-KRAB (CRISPRi) systems is being used in preclinical models to reversibly increase *BDNF* expression in the dentate gyrus [169]. By modifying chromatin at risk-related promoters, these techniques aim to stabilize neural oscillations and enhance neuroplasticity, potentially addressing the cyclic mood episodes characteristic of the disorder [170].

### 4.3. Parkinson’s Disease: Precision Correction of LRRK2 and SNCA

Parkinson’s disease is a heterogeneous neurodegenerative disorder, with about 85% of cases sporadic and ~15% from monogenic or familial causes. Monogenic forms, linked to genes such as SNCA, LRRK2, VPS35, GBA1, PRKN, PINK1, and DJ-1, have helped uncover pathogenic mechanisms, including α-synuclein aggregation, lysosomal-autophagic dysfunction, mitochondrial dysfunction, and synaptic dysregulation [30,32,33]. Recent studies show that both sporadic and genetic PD share neuronal phenotypes in patient-derived dopaminergic neurons, particularly altered synaptic activity and ECM dysregulation [30]. While sporadic and GBA1-associated PD have reduced neuronal activity, PINK1/PRKN mutations can increase synaptic activity, indicating genotype-specific differences [33]. Findings also revealed TMEM16F as a regulator of α-synuclein secretion and spread, linking membrane dynamics to disease progression [171]. Early neuronal abnormalities before neurodegeneration are crucial targets for understanding PD and developing treatments.

Preclinical efforts in PD have advanced toward the precise repair of familial mutations and the mitigation of toxic protein aggregation. ABEs have been successfully deployed in patient-derived induced pluripotent stem cells (iPSCs) to revert the common *LRRK2 G2019S* mutation (adenine to guanine). This single-nucleotide correction normalized kinase activity and enhanced the survival of midbrain dopaminergic neurons [172]. Beyond transition mutations, in human iPSC models, PE has demonstrated the ability to correct the *SNCA A30P* and *A53T* mutations with high fidelity [173]. By utilizing a prime editing guide RNA (pegRNA) to direct a reverse transcriptase to the target site, this technology achieves precise sequence restoration of 60% in hPSCs, while minimizing unintended indels and off-target activity to less than 0.5%, compared to 19.6% with traditional CRISPR-Cas9-mediated repair [173].

### 4.4. Alternating Hemiplegia of Childhood (AHC): Precision Correction of ATP1A3

In a significant preclinical breakthrough for neurogenetic disorders, researchers successfully utilized PE to rescue the phenotypic manifestations of AHC in murine models. By targeting the *ATP1A3* gene, which encodes the alpha-3 subunit of the Na+/K+-ATPase pump, the study used PE to correct the most common pathogenic missense mutations (such as D801N) that cause severe motor and cognitive deficits. Using an engineered AAV-mediated dual-vector system delivered via intracranial injection, the PE achieved high-fidelity sequence restoration in neurons across critical motor circuits, without requiring DSBs. This molecular correction led to a robust recovery of ATPase enzymatic activity, a significant reduction in the frequency and severity of hemiplegic episodes, and a marked improvement in overall motor coordination and survival rates. This study represents a landmark proof-of-concept for the use of in vivo PE to treat dominant-negative neurodevelopmental disorders by precisely repairing point mutations within the endogenous genomic context [174].

### 4.5. Epilepsy: Dravet Syndrome—Correction of SCN8A

In a significant advancement for the treatment of severe genetic epilepsies, recent studies have demonstrated the efficacy of in vivo BE to mitigate the phenotype of SCN8A-related epilepsy in a murine model. Utilizing an ABE delivered via a dual-AAV9 vector system, the researchers targeted the gain-of-function *SCN8A R1872W* mutation, achieving precise reversion in approximately 10% of hippocampal and cortical neurons. This molecular correction led to a robust reduction in spontaneous seizure frequency and a significant delay in the onset of status epilepticus [175]. Notably, while the intervention successfully attenuated paroxysmal electrical activity and improved motor coordination, the current editing efficiency, likely limited by the postmitotic nature of the target neurons and constraints of systemic AAV delivery, was insufficient to substantially extend the subjects’ overall lifespan. This study serves as a critical proof-of-concept for the use of non-DSB-dependent genome surgery to treat early-onset encephalopathies, while highlighting the ongoing need for enhanced delivery vectors to achieve the transduction thresholds required for full phenotypic rescue.

An additional preclinical study targeting SCN2A-related severe epilepsy has demonstrated the therapeutic potential of CRISPRa to restore gene expression in cases of haploinsufficiency. SCN2A encodes the voltage-gated sodium channel Nav1.2, which is critical for action potential initiation and neuronal excitability, and loss-of-function mutations are strongly associated with early-onset epileptic encephalopathy and neurodevelopmental delay [176,177]. In preclinical hESC-derived neurons and mouse models, CRISPRa-mediated upregulation of SCN2A has been shown to increase Nav1.2 expression and partially restore sodium currents and neuronal firing properties [178].

**Table 2:** Preclinical Progress.

Disorder	Target Gene	Editing Tool	Key Advancement	Reference
ASD	*MEF2C*	Base Editing (AeCBE)	Correction of c.104T>C, p.L35P in vivo in mice	[164]
ASD	*CHD8*	CRISPRa	Increased protein levels in iPSC-derived neurons and brain organoids	[167]
Bipolar Disorder	*BDNF*	CRISPRa	Increased protein levels in diverse brain structures	[169]
Parkinson’s disease	*LRRK2*	ABE	Correction of *G2019S* in human iPSCs	[172]
Parkinson’s disease	*SNCA*	Prime Editing	Correction of *A53T* mutation with high fidelity	[173]
Alternating hemiplegia	*ATP1A3*	Prime Editing	Repaired 5 different mutations D801N, E815K, L839P, and G947R (two variants) in AHC patient cells	[174]
Dravet Syndrome	*SCN8A*	ABE	Correction of the *R1872W* mutation in mice model	[175]
Epilepsy and ASD	*SCN2A*	CRISPRa	Increased protein levels in iPSC-derived neurons and mouse model	[178]

## 5. Regulatory and Commercial Landscapes

### 5.1. FDA Guidance and Regulatory Frameworks

The U.S. Food and Drug Administration (FDA) significantly updated its regulatory roadmap in early 2026 to accommodate high-precision modalities such as genome editing [179]. Current FDA Guidance emphasizes a risk-based approach for preclinical testing, requiring sponsors to perform unbiased off-target discovery (e.g., GUIDE-seq) to ensure genomic integrity before human translation. Dose selection strategies have shifted toward utilizing pharmacokinetic and pharmacodynamic (PK/PD) modeling from relevant animal models, such as non-human primates, for CNS delivery, to determine the “minimal effective dose” and mitigate systemic toxicity [180]. For clinical trial design, the agency now favors innovative, accelerated pathways for bespoke therapies in small populations, allowing for adaptive master protocols and the use of natural history data as external controls to facilitate the approval of personalized gene-editing products [179,181].

### 5.2. The FDA’s Shift to Human Models from Animal Models

Recent regulatory and scientific developments have accelerated the shift in drug development from traditional animal models to human-relevant systems such as organoids and advanced in vitro platforms. In 2022, the FDA Modernization Act 2.0 officially eliminated the need for animal testing in drug development, allowing alternative approaches such as human cell-based assays, organoids, and microphysiological systems (organ-on-chip technologies) for preclinical safety and efficacy assessments [182]. This change reflects increasing awareness that animal models often do not accurately predict human responses, especially for complex neurological and genetic diseases, whereas human-derived organoids can better mimic tissue structure, cellular diversity, and disease-specific characteristics [183]. As a result, brain and cortical organoids are being used more to study neurodevelopmental disorders, test gene-editing methods, and evaluate treatments in a patient-specific setting. The FDA has also supported this shift through initiatives promoting New Approach Methodologies (NAMs), which highlight their potential to enhance translational relevance, reduce costs, and address ethical issues associated with animal testing [184]. These advancements collectively represent a major shift toward more predictive, human-centric preclinical models in biomedical research and drug development.

This transition has notable drawbacks and technical challenges. While organoids are highly advanced, they still lack the systemic complexity of living organisms, such as integrated immune responses, full vascular networks, and inter-organ communication found in animal models. This makes it hard to predict how a drug will affect the entire body [185,186,187]. The field also faces issues with standardization and reproducibility, as differences in cell lines and lab protocols can result in inconsistent outcomes, hindering large-scale validation [187,188]. Additionally, a regulatory gap exists, with researchers and agencies working to develop new safety standards that rival the rigorous benchmarks established by animal studies over decades [189]. Despite these obstacles, moving toward human-centric models is a significant step forward in producing more predictive biomedical research, helping to bridge the gap between preclinical discovery and clinical application [190].

### 5.3. The Commercial Pipeline and Strategic Partnerships

The commercial success of gene therapy increasingly depends on biotech-pharma collaborations that bridge the gap between preclinical research and major human trials [191]. Over the past five years (2021–2025), the industry has experienced substantial capital flow; after hitting a peak of $22.7 billion in 2021, yearly investments stabilized around $11.7 billion to $12.6 billion during 2023–2024, then rose again in late 2025 due to a “tale of haves and have-nots,” where funding favored late-stage assets. Large companies are shifting toward partnerships, offering manufacturing and regulatory support to move assets from academia into Phase 3 trials [191,192]. This trend is evident in the growing global pipeline: by late 2025, over 2000 clinical trials will be underway in cell and gene therapy (CGT), with gene therapies making up about 49% of development projects [193]. The neurology sector has seen notable growth, with a 22.5% Compound Annual Growth Rate (CAGR), and is now a key focus alongside oncology and rare diseases [194]. In 2025–2026, the market experienced a rise in strategic deals, such as the $5.8 billion increase in Bristol Myers Squibb’s portfolio and collaborations between Neurocrine, Voyager, and Intellia, mainly targeting IV-delivered neuro gene therapies and non-viral platforms [195]. These collaborations are essential for increasing Good Manufacturing Practice (GMP) production capacity and meeting clinical needs for worldwide commercialization, particularly since the neurological gene therapy sector is dominating the market in 2023 and is expected to continue to do so through 2032 [196].

## 6. Future Perspectives and Personalized Medicine

### 6.1. Biomarkers for Patient Stratification and Monitoring

The effectiveness of gene therapy for neurological and psychiatric disorders will increasingly rely on identifying and validating reliable biomarkers. These biomarkers are essential for selecting appropriate patients, forecasting how they will respond to treatment, and tracking treatment progress. In CNS diseases, biomarkers are especially important because clinical symptoms often appear only after significant neuronal damage. Sensitive molecular markers can enable earlier intervention and help optimize the timing of therapies [197].

Neurofilament light chain (NfL) is one of the most promising biomarkers in neurology [197]. It is a structural protein released into cerebrospinal fluid and blood after axonal damage. Elevated NfL levels are observed in several neurodegenerative diseases, such as AD, PD, ALS, and multiple sclerosis [198]. Longitudinal studies indicate that NfL levels correlate with disease progression and treatment response, making it a useful tool for assessing therapeutic outcomes in clinical trials. In gene therapy research, NfL can act as an early marker of neuroprotection or neuronal rescue, often before clinical improvements appear. In addition to NfL, new biomarkers, such as phosphorylated tau, synaptic proteins, and transcriptomic signatures, are being investigated to improve patient stratification and support personalized therapy choices [197].

### 6.2. Artificial Intelligence and Systems Biology

The fusion of artificial intelligence (AI) and systems biology is set to be crucial in developing the next generation of gene therapies [199]. Progress in machine learning enables the analysis of large biological datasets generated by multi-omics methods, such as genomics, transcriptomics, proteomics, metabolomics, and epigenomics [200,201,202,203,204]. By combining these diverse datasets, AI-based strategies can identify intricate molecular network changes underlying neurological and psychiatric disorders [205,206].

In gene therapy, AI models are already being used to develop high-precision genome editors that offer greater specificity and fewer off-target effects [207]. These computational tools can forecast guide RNA effectiveness, find potential off-target regions throughout the genome, and refine vector design to improve delivery success [208]. Additionally, AI-driven protein engineering is accelerating the development of new Cas variants, base editors, and prime editors with greater accuracy and broader targeting capabilities [209]. Systems biology approaches further help uncover patient-specific disease mechanisms by linking genetic variants to cellular pathways and regulatory networks. This approach supports the development of personalized gene-editing treatments tailored to an individual’s genetic makeup. Such precision strategies could be especially beneficial for complex neurological and psychiatric conditions, where disease symptoms often result from complex interactions among multiple genes and environmental influences [210].

### 6.3. Toward Durable, One-Time Therapies

Looking ahead, gene therapy could significantly change how neurological and psychiatric disorders are treated. Unlike traditional drugs, which require ongoing use and primarily target downstream processes, gene therapies focus on the root genetic causes. Since neurons are mostly post-mitotic, effective genome editing in these cells can yield long-lasting or even lifelong benefits after a single treatment [28].

Progress in delivery systems, genome editing, and molecular diagnostics is steadily making this vision more achievable. Enhancements in both viral and non-viral vectors enable more efficient, targeted delivery to the brain [35,93,138,139,140,141,142,143]. Meanwhile, advanced editing platforms such as BE and PE offer safer, more accurate ways to correct disease-causing mutations [40,43,164,168,175,211]. Additionally, the combination of biomarker-guided therapies and AI-powered design is setting the stage for highly personalized gene treatments [197,205].

While major challenges such as long-term safety, effective brain-wide delivery, and ethical issues persist, the convergence of gene editing, advanced delivery systems, and precision medicine offers extraordinary possibilities. These combined innovations could eventually provide lasting, one-time cures for many neurological and psychiatric disorders that were once considered incurable, representing a revolutionary change in treating brain diseases.

## 7. Critical Unknowns in Clinical Implementation

As gene editing advances toward clinical use for complex neurological and psychiatric disorders, important questions about the best scale and precision of these interventions remain. Resolving these issues is crucial for establishing the therapeutic window and achieving long-term success.

### 7.1. The Challenge of Polygenic Architecture and Network Modulation

A major unknown in treating disorders like AD and PD is that they rarely result from a single genetic mutation. Instead, they are shaped by a complex system of multiple risk alleles [212]. When a disease is influenced by a network of genetic variants, it raises a key question: how many targets need to be altered to achieve clinical benefit? Current approaches mainly target key “hubs,” such as the APOE ε4 allele in AD or GBA1 mutations in PD, based on the idea that altering a central driver can help stabilize the entire disease network [30,161]. But it is still unclear whether editing multiple loci at once will be required to produce a significant effect, and whether doing so would greatly increase the risk of off-target effects and chromosomal translocations.

### 7.2. Determining Optimal Cellular Specificity and Circuit Targeting

Another critical uncertainty concerns the scope of cellular-level intervention: should the therapy target all cells within a brain region, or be limited to specific cell types? In many neurological disorders, the pathology is circuit-specific, such as dopaminergic neurons in the substantia nigra for PD. However, glia also play a significant role in neuroinflammation and protein clearance [213,214]. A key challenge in implementation is deciding whether to use broad, pan-neuronal expression, which risks impacting healthy circuits, or to pursue high cellular specificity with engineered viral capsids, which might overlook non-neuronal contributors to the disease.

### 7.3. Establishing Efficacy Thresholds and Therapeutic Dosing

Closely tied to specificity is the uncertainty about the need for cellular coverage: must we target every relevant cell in the region, or is targeting a small number enough? Some lysosomal storage disorders exhibit a “cross-correction” effect, where a few edited cells secrete enzymes that help nearby cells [215]. But for cell-autonomous psychiatric conditions or intracellular proteinopathies, the requirements might be more stringent [216]. We still lack a clear understanding of the clinical threshold, whether a 10% correction is too low to make a difference, or if it can tip the neurobiological balance toward health [217]. Determining these dose–response relationships is essential for advancing these therapies into human trials.

## 8. Conclusions

Neurological and psychiatric disorders constitute a global health crisis, affecting around 3.4 billion people and being the leading cause of disability worldwide. The research paradigm has shifted from traditional symptom management to disease-modifying approaches targeting the underlying molecular causes. This change is fueled by high-precision genome-editing technologies such as base editing and prime editing, which are well-suited to the non-dividing environment of the CNS. Since neurons do not divide, targeted genomic corrections promise durable, single-treatment effects that can last a lifetime.

The clinical application of these therapies depends heavily on overcoming the BBB, the main obstacle to CNS delivery. Advances such as engineered AAV capsids with improved targeting, AAVLINK systems for larger cargo, and stem cell-derived exosomes capable of crossing the BBB are making progress. The upcoming 2025 FDA approval of Itvisma for spinal muscular atrophy, along with ongoing trials for Parkinson’s and Huntington’s diseases, indicates that gene-targeted therapies are transitioning from concept to reality.

In the future, integrating artificial intelligence and systems biology will enhance the design of more precise, patient-specific editors. Human-centric preclinical models, such as iPSC-derived organoids, will improve safety and efficacy predictions. Although challenges remain in brain-wide delivery and long-term safety, the combination of precise editing, advanced delivery methods, and biomarker-guided monitoring opens exciting possibilities. These synergistic advances are paving the way for a new era in medicine, where previously incurable brain diseases could be treated with one-time, lasting cures.

## Data Availability

Not applicable.

## Abbreviations

The following abbreviations are used in this manuscript:

AADC	Aromatic L-amino acid decarboxylase
AAV	Adeno-Associated Virus
ABE	Adenine Base Editor
AD	Alzheimer’s Disease
AdV	Adenoviral Vector
AHC	Alternating Hemiplegia of Childhood
AI	Artificial Intelligence
ALS	Amyotrophic Lateral Sclerosis
ASD	Autism Spectrum Disorder
ASO	Antisense Oligonucleotide
BBB	Blood–Brain Barrier
BD	Bipolar Disorder
BDNF	Brain-Derived Neurotrophic Factor
BE	Base Editing
BLA	Biologics License Application
CAGR	Compound Annual Growth Rate
CED	Convection-Enhanced Delivery
CGT	Cell and Gene Therapy
CMV	Cytomegalovirus
CNS	Central Nervous System
CRISPR	Clustered Regularly Interspaced Short Palindromic Repeats
CRISPRa	CRISPR activation
CRISPRi	CRISPR interference
DALY	Disability-Adjusted Life Year
dCas9	Catalytically inactive “dead” Cas9
DNA	Deoxyribonucleic acid
DSB	Double-Strand Break
ECM	Extracellular Matrix
EV	Extracellular Vesicle
FDA	U.S. Food and Drug Administration
FUS	Focused Ultrasound
GDNF	Glial Cell Line-Derived Neurotrophic Factor
GMP	Good Manufacturing Practice
HD	Huntington’s Disease
HDR	Homology-Directed Repair
HTT	Huntingtin
Indel	Insertion or deletion
iPSC	Induced Pluripotent Stem Cell
kb	Kilobase
LNP	Lipid Nanoparticle
MCI	Mild Cognitive Impairment
MENP	Magneto-electric Nanoparticle
miRNA	MicroRNA
mRNA	Messenger RNA
MSC	Mesenchymal Stem Cell
NAM	New Approach Methodology
NBS	Newborn Screening
NfL	Neurofilament light chain
NHEJ	Non-Homologous End Joining
PAM	Protospacer Adjacent Motif
PD	Parkinson’s Disease
PE	Prime Editing
pegRNA	Prime editing guide RNA
PK/PD	Pharmacokinetic and pharmacodynamic
PNP	Polymeric Nanoparticle
RIDE	Ribonucleoprotein Delivery system
RISC	RNA-induced silencing complex
RNA	Ribonucleic acid
RNP	Ribonucleoprotein
RT	Reverse Transcriptase
RVD	Repeat-variable diresidue
sgRNA	Single-guide RNA
siRNA	Small interfering RNA
SMA	Spinal Muscular Atrophy
TALEN	Transcription Activator-Like Effector Nuclease
